# Annotations

**Published:** 1898-10-22

**Authors:** 


### ANNOTATIONS.

### Diphtheria Antitoxin.

ACCORDING to the report of Dr. Sims Woodhead, director of the Research Laboratories of the Royal Colleges of Physicians and Surgeons, in addition to preparing antitoxic serum for the treatment of diphtheria in the hospitals of the Metropolitan Asylums Board, arrangements have been made for testing any serum sent for examination, and it is satisfactory to note that all the samples sent by the two firms which have availed themselves of this privilege have proved to be of excellent strength and quality. Special attention has been given to the question of the standardisation of antitoxin, and so successful have the new methods of preparation which are now in operation proved that the average strength of the serum produced at the laboratory has been found to be 375 antitoxin units per c.c. It is especially satisfactory to note that a large amount of antitoxin, namely, 2,003 doses, has been supplied to various general and children's hospitals in London during the past year, as well as to certain medical practitioners, for cases "fulfilling the necessary conditions." We cannot but feel, however, that it would be well if these conditions were somewhat relaxed, and if some means could be adopted by which medical practitioners could obtain such free and immediate access to antitoxin that this valuable drug should be available for all cases of diphtheria at the earliest possible stage. This is a matter of very considerable importance. The whole teaching of the experience which has been gained during the past three years in the hospitals of the Metropolitan Asylums Board is to the effect that the early and the free use of antitoxin cuts short diphtheria in a remarkable manner. Everything seems to depend upon early administration, and, in view of the obvious failure of the attempts which have been made at such cost to stamp out diphtheria by isolation, it would seem that as a matter of public policy every effort should be made to bring antitoxin to the homes of the poor at the very earliest possible moment. Rich and poor alike can get the benefit of this antidote, free gratis and for nothing, when once they get into the hospitals, and it seems only reasonable to ask that every facility should be afforded for its administration on the very first discovery of the disease. There are many delays in removal to hospital; there need be none in administrating an antidote.

### Specialists and Practitioners.

AN extraordinary suggestion reaches us from America as to the relations which should exist between the specialist and the general practitioner. It is Dr. Melville Black, of Denver, who makes it in the *Colorado Medical Journal*, so as yet the innovation is a long way off; still, it is as well to know beforehand what we are to come to. The suggestion, shortly put, is this—that when a practitioner refers a case to a specialist he should in return receive from the specialist a fee; the idea being that in so referring a case the practitioner is not rendering "actual professional services," and so will not be paid for his advice by the patient. Dr. Black says that when a general practitioner sees fit to send certain cases to a specialist "he displays a magnanimity unparalleled in other fields of labour," and he goes on to say that when the bill for his services is presented "no attention is paid to it, and if payment is pressed, the next time a physician is needed someone else is called in. Now who is to compensate this physician? There is but one answer—the specialist. If the specialist does not pay him for the time expended no one else does." All of which we think is very absurd. In the first place, it does not matter a fig whether a practitioner orders his patient to take a pill, or to rub on a liniment, or to go to a specialist. In each case he has exercised his professional discretion and skill, and in each case he is equally entitled to be paid for his services. But outside and beyond all that comes the ethical side of the question, and we protest utterly and entirely against the suggestion that in the choice of a specialist the question of gain to the practitioner should in any way arise. In every case the practitioner is bound to use his best skill for the patient's greatest benefit. He has no right to take fees from specialists any more than he has to take Christmas geese from dealers in meat juices and medicated wines.

### "Massage" and Immorality.

WE do not wish to comment on the filthy details which were laid before Mr. Curtis Bennett at the Marylebone Police Court last Saturday in the so-called "massage" case, but we must express our satisfaction that it has at last been found possible to mete out some punishment to one at least of the scoundrels who, under the name of massage establishments, not only keep up, but actually advertise what are in reality dens of iniquity, and who, in the guise of nurses and under the pretence of carrying out a pseudo-medical treatment, use women for the vilest purposes. The real point of interest in the case is the public demonstration of what everyone who knows anything has known for long, namely, the real character of these advertising massage shops and the delusive character of their advertisements. What could be more innocent in appearance than the following: "Balneopathic institution for the treatment of rheumatism, gout, sciatica, and neuralgia by dry hot-air baths, massage, and discipline, &c."? And yet what more vile than the reality! One word alone shows the nature of the establishment, but that is a word which might easily be overlooked by the innocent or the careless; and those who know, as we do, what a number of more or less wealthy people there are who are ready to try any form of treatment for their imaginary ailments, know what a field they offer for exploitation by the bully and the blackmailer. The advertisements which appear in certain papers and disgrace the streets in which strangers and the wealthy idle mostly congregate do infinite harm in every way. To those of vicious tendencies, but ignorant of vice's ways, they serve as an introduction to a downward course, while to the well-to-do and respectable *malade imaginaire* they are mere traps set by the blackmailer. Why they should have been permitted for so long, and why, under the name of "massage," disreputable persons masquerading as "nurses" should have been allowed so openly to ply such a trade is a mystery. English liberty, however, is a cloak for much that is evil.

